# A role for *Galgt1* in skeletal muscle regeneration

**DOI:** 10.1186/s13395-014-0028-0

**Published:** 2015-01-27

**Authors:** Neha Singhal, Paul T Martin

**Affiliations:** Center for Gene Therapy, The Research Institute at Nationwide Children’s Hospital, Columbus, USA; Department of Pediatrics, The Ohio State University College of Medicine, 700 Children’s Drive, Columbus, OH 43205 USA; Department of Physiology and Cell Biology, The Ohio State University College of Medicine, 700 Children’s Drive, Columbus, OH 43205 USA

**Keywords:** Duchenne muscular dystrophy, Muscle regeneration, Ganglioside, Satellite cell, Apoptosis

## Abstract

**Background:**

Cell surface glycans are known to play vital roles in muscle membrane stability and muscle disease, but to date, roles for glycans in muscle regeneration have been less well understood. Here, we describe a role for complex gangliosides synthesized by the *Galgt1* gene in muscle regeneration.

**Methods:**

Cardiotoxin-injected wild type (WT) and *Galgt1*^*−/−*^ muscles, and *mdx* and *Galgt1*^*−/−*^*mdx* muscles, were used to study regeneration in response to acute and chronic injury, respectively. Muscle tissue was analyzed at various time points for morphometric measurements and for gene expression changes in satellite cell and muscle differentiation markers by quantitative real-time polymerase chain reaction (qRT-PCR). Primary cell cultures were used to measure growth rate and myotube formation and to identify *Galgt1* expression changes after cardiotoxin by fluorescence-activated cell sorting (FACS). Primary cell culture and tissue sections were also used to quantify satellite cell apoptosis.

**Results:**

A query of a microarray data set of cardiotoxin-induced mouse muscle gene expression changes identified *Galgt1* as the most upregulated glycosylation gene immediately after muscle injury. This was validated by qRT-PCR as a 23-fold upregulation in *Galgt1* expression 1 day after cardiotoxin administration and a 16-fold upregulation in 6-week-old *mdx* muscles. These changes correlated with increased expression of Galgt1 protein and GM1 ganglioside in mononuclear muscle cells. In the absence of *Galgt1*, cardiotoxin-induced injury led to significantly reduced myofiber diameters after 14 and 28 days of regeneration. Myofiber diameters were also significantly reduced in *Galgt1*-deficient *mdx* mice compared to age-matched *mdx* controls, and this was coupled with a significant increase in the loss of muscle tissue. Cardiotoxin-injected *Galgt1*^*−/−*^ muscles showed reduced gene expression of the satellite cell marker Pax7 and increased expression of myoblast markers MyoD, Myf5, and Myogenin after injury along with a tenfold increase in apoptosis of Pax7-positive muscle cells. Cultured primary *Galgt1*^*−/−*^ muscle cells showed a normal growth rate but demonstrated premature fusion into myofibers, resulting in an overall impairment of myofiber formation coupled with a threefold increase in muscle cell apoptosis.

**Conclusions:**

These experiments demonstrate a role for *Galgt1* in skeletal muscle regeneration and suggest that complex gangliosides made by *Galgt1* modulate the survival and differentiation of satellite cells.

## Background

Skeletal muscle displays a remarkable potential to regenerate after injury, and satellite cells, the predominant stem cells in adult skeletal muscle, play an essential role in this process [1-3]. Satellite cells normally reside in the niche between the skeletal myofiber membrane and its surrounding basal lamina [1], which is composed predominantly of laminin 211 (α2, β1, γ1) and collagen IV (α1, α2) [4-8]. When a myofiber is injured beyond repair due to membrane rupture, mechanical insult, or aberrant calcium homeostasis, the resulting necrosis of skeletal myofibers is coupled with myofiber clearance by invading immune cells and the stimulation of asymmetric satellite cell division to generate myoblasts that will ultimately fuse to form new skeletal myofibers and new satellite cells that will replenish the muscle stem cell niche. In response to acute injury, newly regenerated contractile skeletal myofibers are formed over a period of about 2 weeks [2,9-12]. Certain compounds, including the snake peptide cardiotoxin (CTX), can mimic the aberrant calcium homeostasis that occurs during muscle damage and be used to synchronize the induction of muscle regeneration [13,14].

In the muscular dystrophies, impaired regeneration, coupled with chronic muscle damage, can lead to muscle wasting, which involves the loss of muscle tissue and its replacement with extracellular matrix or fat [15]. Muscle wasting in the muscular dystrophies drives the development of muscle weakness and ultimately death. Muscle wasting, which likely occurs due to an imbalance between muscle degeneration and regeneration, is a hallmark of Duchenne muscular dystrophy (DMD). DMD is caused by mutations or deletions in the dystrophin (*DMD*) gene that give rise to an absence of dystrophin protein expression in skeletal and cardiac muscle [16,17]. The most commonly used DMD animal model is the *mdx* mouse [18-20]. The *mdx* mouse muscle undergoes chronic cycles of degeneration coupled with muscle regeneration. These cycles begin at about 3 weeks of age and peak at 4–6 weeks of age, when a severe period of muscle damage occurs. This is followed by a more subdued, but chronic, disease process throughout the remainder of the mouse’s lifespan [21]. Introduction of secondary gene deletion or transgene overexpression has implicated a number of additional genes, including utrophin, telomerase, integrin α7, sarcospan, *Galgt2*, and *Cmah*, amongst others, as modulators of mdx disease severity [22-32].

The important cellular mechanisms involved in muscle regeneration, satellite cell activation and differentiation, myoblast migration and fusion, and myofiber growth are mediated by signaling molecules including Akt and Wnt kinases, mTOR, Notch receptors, and their down-stream signals [33-37]. While at least some cell signaling processes at the membrane involved in muscle regeneration are well described, roles for cell surface glycans, with the exception of syndecan proteoglycans and glycosaminoglycan endosulfatases [38-44], remain poorly understood. This stands in contrast with the large number of studies demonstrating roles for glycans in mediating muscle membrane stability and causing muscle disease [31,32,45-47]. Complex gangliosides are good candidates for mediating aspects of muscle regeneration. Gangliosides have been shown to be able to modulate cell-cell interactions, cell signal transduction, and the activity of membrane ion channels in numerous cell types, including myoblasts [48-55]. GM2, GD2, and all down-stream complex gangliosides, including GM1, require the enzymatic activity encoded by the *Galgt1* gene for their production [56,57]. *Galgt1* has clear roles in mediating the binding of complex gangliosides to endogenous sialic acid-binding lectins that are known to control important aspects of nervous system development, for example axon guidance, axon stability, and axonal regeneration [53,57-60]. In this study, we demonstrate dynamic and pronounced changes in *Galgt1* expression during skeletal muscle regeneration and demonstrate a role for this gene in the regeneration process.

## Methods

### Materials

Anti-ganglioside GM1 antibody was purchased from Millipore (345757). Rabbit polyclonal antibody to Galgt1 peptide CQVRAVDLTKAFDAEE was made in our lab by immunizing rabbits with KLH-conjugated peptide, after which antibody was purified over peptide-conjugated resin as previously described [61]. Anti-mouse Pax7 antibody was a gift from Dr. Michael Rudnicki (Ottawa Health Research Institute). Anti-mouse integrin α7 conjugated to fluorescein isothiocyanate (FITC) was purchased from MBL International (K0046-4) and R & D Systems (FAB3518F). Anti-mouse CD11b conjugated to FITC and Rat anti-Ertr7 were gifts from Dr. Jill Rafael-Fortney (The Ohio State University). Rat anti-mouse Ly-6A/E conjugated to FITC (Sca1, 553335), rat anti-mouse CD45 conjugated to PE-Cy7 (552848), rat anti-mouse CD31 conjugated to APC (551262), and rat anti-mouse CD16/CD32 Fc block (553142) were purchased from BD Biosciences. All secondary antibodies conjugated to fluorophores were purchased from Jackson ImmunoResearch. Rhodamine-conjugated α-bungarotoxin was purchased from Life Technologies. Sections from normal human and Duchenne muscular dystrophy muscle biopsies from clinical specimens archived as part of the United Dystrophinopathy Project were obtained in accordance with approval from the Institutional Review Board.

### Mice

All animal experiments were conducted after approval from the Institutional Animal Use and Care Committee (IACUC) at The Research Institute at Nationwide Children’s Hospital. Mice lacking *Galgt1* (*Galgt1*^*−/−*^) were obtained from Consortium for Functional Glycomics (www.functionalglycomics.org) and were originally made by Proia and colleagues [57,62]. *mdx* and wild type (C57Bl/6) mice were purchased from Jackson Laboratories. *Galgt1*^*−/−*^*mdx* mice were obtained by interbreeding of *Galgt1*^*−/−*^ mice with *mdx* mice. Six-week-old, 3-month-old, and 6-month-old animals were used for wild type (WT), *Galgt1*^*−/−*^, *mdx*, and *Galgt1*^*−/−*^*mdx* experiments as indicated.

### Cardiotoxin-induced muscle regeneration

Two-month-old animals were used for cardiotoxin injection experiments. Cardiotoxin, from *Naja mossambica mossambica* venom, was purchased from Sigma-Aldrich (C9759). It was diluted to a 10-μM concentration in phosphate-buffered saline (PBS) and injected intramuscularly into the gastrocnemius, tibialis anterior, or quadriceps muscles in a volume of 50 μl (gastroc or quad) or 25 μl (tibialis anterior (TA)). Muscles were collected 1, 4, 7, 14, and 28 days after cardiotoxin injection, snap frozen in liquid nitrogen-cooled isopentane, and sectioned at 8 μm in cross section on a cryostat for histological analysis. Mock-injected muscles from control animals were harvested at these same time points as controls.

### Histology

Muscles were dissected from tendon to tendon, mounted on OCT, and snap frozen in liquid nitrogen-cooled isopentane. Muscles were cross-sectioned on a cryostat at 8-μm thickness and mounted on slides for staining. For immunostaining, sections were blocked in 3 mg/ml bovine serum albumin (BSA) in PBS. Primary antibodies were diluted in 3 mg/ml BSA/PBS and sections incubated overnight at 4°C. After washing in PBS, sections were stained with a secondary antibody, added at a dilution of 1:250, for 1 h. After washing, slides were mounted in Prolong Gold Antifade with 4’,6-diamidino-2-phenylindole (DAPI) and visualized on a Zeiss Axiophot epifluorescence microscope using fluorescein-, rhodamine-, or DAPI-specific optics. For hematoxylin and eosin (H & E) staining, cryostat-cut 8-μm muscle cross sections were first fixed in 10% neutral-buffered formalin. After washing in tap water, slides were stained with Gill’s 3 Hematoxylin for 2 min, washed in tap water, then bluing agent, and again in tap water. Sections were then stained in eosin for 1 min and excess stain removed by dipping in 30% ethanol, after which the sections were dehydrated in 70%, 90%, 95%, and 100% ethanol. The sections were cleared in xylene and mounted in the xylene-based mounting media Cytoseal.

### Quantification of muscle morphometry

Myofiber diameter and central nuclei measurements were carried out and quantified using Zeiss AxioVision Rel.4.8 software as previously described [32]. H & E images were photographed at × 20 magnification for such measurements. Briefly, muscle sections from at least four separate planes of the injected muscle were counted in total and averaged to create single average data points for each measure, and the average of these measures from all animals per time point or condition was then used to determine the average myofiber diameter, the percentage of myofibers with central nuclei, the number of myofibers per unit area, or the percentage of non-muscle area. For CTX experiments comparing wild type and *Galgt1*^*−/−*^ muscles, and for experiments comparing *mdx* and *Galgt1*^*−/−*^*mdx* muscles, six muscles were averaged per condition, with 1,600–3,600 total myofibers counted per condition.

### Fluorescence Activated Cell Sorting (FACS)

The limb muscles of three WT mice were injected with 10-μM cardiotoxin and compared to WT animals injected with an equivalent volume of PBS. One day after injection, the muscles were removed, minced, and digested with 5 mg/ml collagenase IV and 1.2 U/ml Dispase for 30 min at 37°C. The reaction was stopped by dilution with DMEM, filtration of cells through a 70-μm membrane and then a 40-μm cell strainer, and centrifugation in horse serum to remove enzymes from the cells. Cells were stained after washing in 0.5% BSA/PBS. Cells were blocked with Mouse BD Fc Block (BD Pharmingen 553141) at a concentration of 2 μl/10^6^ cells on ice for 10 min followed by staining with fluorophore-conjugated primary antibody at a concentration of 2 μl/10^6^ cells for 10 min on ice. Cells were then washed, resuspended, and filtered in fluorescence-activated cell sorting (FACS) tubes. Propidium iodide (PI, 2 μg/ml) was added just before sorting to identify dead cells. Cells were first gated based on side scatter and forward scatter then based on the height and width. PI-positive (dead) cells were removed prior to further analysis. Cells were sorted for Sca1^+^ and Sca1^−^ fractions and each fraction was further sorted for CD31^+^, CD45^+^, and CD31^−^CD45^−^. Fractions were collected and analyzed for *Galgt1* mRNA using quantitative real-time polymerase chain reaction (qRT-PCR) as previously described [63]. FACS analysis was repeated with gating for CD31^−^APC and CD45^−^PE Cy7 and the CD31^−^CD45^−^ fractions, with sorting for integrin α7 against side scatter. The integrin α7^+^CD31^−^CD45^−^, integrin α7^−^CD31^−^CD45^−^, integrin α7^−^CD31^+^CD45^−^, and integrin α7^−^CD31^−^CD45^+^ fractions were collected and analyzed for *Galgt1* mRNA using qRT-PCR as before [63].

### Semi-quantitative real-time polymerase chain reaction (qRT-PCR)

Gastrocnemius muscles were homogenized in TRIzol (Invitrogen) and total RNA was extracted and purified using either RNeasy Mini or Micro kits (Qiagen) according to the manufacturer’s instructions. The quality of RNA was determined by electrophoresis using 6000 Nano LabChip kit on Bioanalyzer 2100 (Agilent). The samples without RNA degradation were used to synthesize cDNA using High Capacity Reverse Transcriptase kit (Applied Biosystems). Taqman ABI 7500 sequence detection system (Applied Biosystems) was used for real-time qPCR measurements on the samples using the delta-delta CT method [64]. qRT-PCR probes for Galgt1 and 18S RNA have been described in previous publications [32,65]. Primers for dystrophin (Mm01216926_m1), Pax7 (Mm00834079_m1), Myf5 (Mm00435125_m1), MyoD (Mm00440387_m1), Myogenin (Mm00446194_m1), and Myh3 (Mm01332463_m1) were purchased from Applied Biosystems. 18S was used as an internal control. Measures shown are replicates of three to four muscles per condition.

### TUNEL staining

Terminal deoxynucleotidyl transferase (TUNEL) staining was performed according to the manufacturer’s instructions using the Click-iT® TUNEL Alexa Fluor® 488 Imaging Assay (Life Technologies, C10245). For Pax7-TUNEL co-staining, TUNEL staining was done prior to Pax7 staining, which was done as previously described [66]. Replicates shown are averages of six muscles per condition for tissue staining or of three to six replicates per condition for cell culture experiments.

### Muscle cell isolation

Gastrocnemius, quadriceps, tibialis anterior (TA), and triceps were dissected from WT and *Galgt1*^*−/−*^ mice as previously described [61]. The skeletal muscles were collected in ice-cold sterile PBS and dissected muscles were minced in a sterile laminar-flow hood using aseptic conditions. The minced muscles were digested in a solution containing 1.2 U/ml Dispase and 5 mg/ml collagenase IV. The minced tissue was incubated with enzymes at 37°C for 30 min with intermittent pipetting every 10 min, followed by addition of DMEM/F12 media + 10% heat-inactivated horse serum + 1% penicillin-streptomycin solution. The solution was then filtered through 70-μm cell strainer and then a 40-μm cell strainer. Cells were then centrifuged at 1,500 rpm at 4°C for 10 min. The cell pellet was resuspended in 0.5% BSA/PBS and overlayed on 100% heat-inactivated horse serum and centrifuged at 220 × *g* at 4°C for 10 min. The pellet was then washed in 0.5% BSA/PBS and centrifuged at 1,500 rpm at 4°C for 10 min and resuspended in growth media (DMEM/F-12 + 10% FBS + 4% chick embryo extract + 1% P/S). Cells were pre-plated on uncoated tissue culture plates, with muscle-cell-enriched supernatant removed after fibroblasts were allowed to adhere for 20 min. The pre-plating step was repeated if needed to obtain pure muscle cell cultures. Purified muscle cells were then plated on 0.1% collagen-1-coated tissue culture plates. As in our previous studies [61], such cultures typically showed an excess of 90% positive staining for c-met, a muscle cell marker.

### Muscle cell growth rate and fusion assays

To measure the growth rate of isolated muscle cells, cells extracted and purified from WT and *Galgt1*^*−/−*^ skeletal muscles were grown in the growth media (DMEM/F-12 + 10% FBS + 4% chick embryo extract + 1% P/S) and were counted at three different time points (1, 2, and 3 days post-plating). For the muscle cell fusion assay, cells were grown in the growth media till they reached 100% confluence and were then switched to fusion media (×1 DMEM/F-12 + 2% horse serum + 1% P/S). Skeletal myofibers were analyzed at four time points—Day 0 (just before addition of fusion media), Day 1 (1 day after addition of fusion media), Day 3, and Day 6. Sixteen equivalent fields of view were evaluated per well with at least three wells evaluated per time point and per genotype. Data shown are averages of six experiments per condition, normalized to baseline cell number on Day 0 in each instance.

### Statistics

All determinations of significance were done by ANOVA with *post hoc* Bonferroni analysis and/or unpaired *t*-tests. A *P* value of less than or equal to 0.05 was considered significant.

## Results

### *Galgt1* and GM1 expression are increased in regenerating skeletal muscle

**Figure 1:**
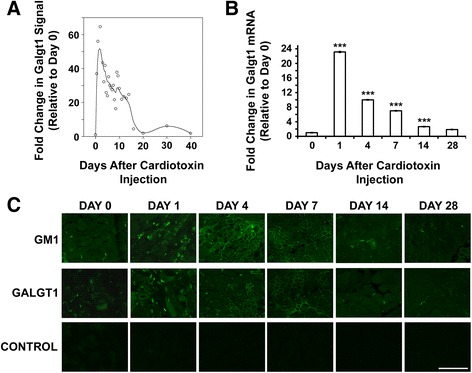
**Expression of** ***Galgt1*** **during cardiotoxin-induced muscle regeneration. (A)** *Galgt1* signal data from the publicly available Children’s National Medical Center Affymetrix microarray study on regenerating mouse muscle after cardiotoxin-induced injury. **(B)** qRT-PCR measurement of the fold change in the expression of *Galgt1* gene in WT muscle 1, 4, 7, 14, or 28 days after cardiotoxin-induced muscle injury. **(C)** Wild type gastrocnemius muscle was stained with antibody to *Galgt1*-dependent complex ganglioside GM1, Galgt1 protein, or with secondary antibody alone (control) after cardiotoxin-induced injury of wild type muscle. Errors in **(B)** are SEM averaged from six muscles per condition with two to three measures per muscle. ***P < 0.001, Bar is 50 μm.

We originally queried the publically available Children’s National Medical Center Affymetrix microarray data set developed by Hoffman and colleagues to assess gene expression changes after CTX-induced muscle regeneration in wild type mice (http://microarray.cnmcresearch.org, now http://pepr.cnmcresearch.org [67,68]). We analyzed changes in the 1,769 glycosylation-related genes listed on the glycan microarray from the Consortium for Functional Glycomics (http://www.functionalglycomics.org), which includes known glycosyltransferase, glycosidase, and glycan metabolism genes, as well as mammalian lectins and some glycoproteins where glycan functions have been described, using this database. Of the glycosylation genes queried, *Galgt1* was the most elevated relative to control, with a greater than 50-fold increase in signal in the first day after CTX treatment that remained elevated for about 14 days (Figure 1A). To validate this finding, we measured levels of *Galgt1* gene expression by qRT-PCR in WT C57Bl/6 mouse muscles injected with CTX at 1, 4, 7, 14, and 28 days post-injection and compared expression to untreated muscle (Day 0) (Figure 1B). *Galgt1* expression was elevated 23-fold at 1 day post-CTX injection, and, much like the microarray data, showed significantly elevated levels in the first 14 days after CTX. The induction of *Galgt1* expression declined as regeneration progressed, nearing baseline again by Day 28, a time at which muscle regeneration is typically complete.

We next stained skeletal muscles taken at different days after CTX injection with an antibody to Galgt1 protein or with an antibody to GM1, a complex ganglioside that requires Galgt1 activity for its expression [69] (Figure 1C). GM1 staining was chosen to reflect Galgt1 activity as antibodies to GM2 and GD2 can also cross-react with glycans made by *Galgt2*, another glycosyltransferase in the *Galgt1* gene family, while antibodies to GM1 do not [69]. As shown previously, antibodies to Galgt1 protein and GM1 ganglioside both stained the neuromuscular junction (NMJ) in control muscle (uninjected Day 0) [63]. This was confirmed by co-staining of NMJs with rhodamine α bungarotoxin (data not shown). On Day 1 after CTX injection, Galgt1 and GM1 staining were both increased on intramuscular mononuclear cells. This staining of mononuclear cells was also present on Day 4 and Day 7, though here, myofiber staining was also evident. By Day 14 and Day 28, both GM1 and Galgt1 staining were primarily localized to regions including the NMJ, just as at Day 0 (Figure 1C). Thus, Galgt1 protein and GM1, a ganglioside product of Galgt1 activity, were both transiently elevated in muscles after CTX addition, much as would be expected given the dramatic increase in *Galgt1* gene expression.

### *Galgt1* and GM1 expression are increased in dystrophic skeletal muscle

**Figure 2:**
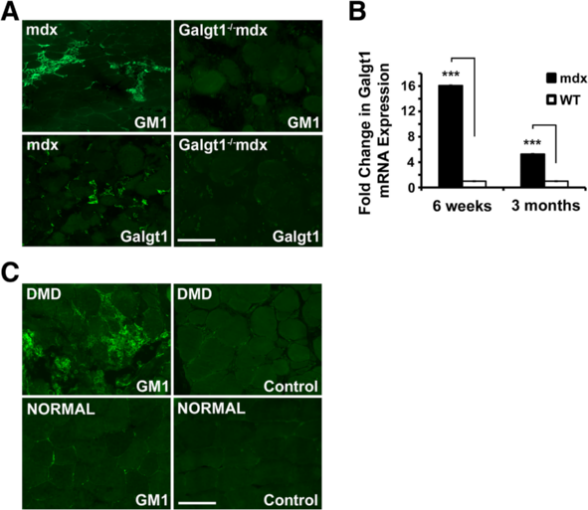
**GM1 and** ***Galgt1*** **expression in dystrophic skeletal muscle. (A)** Immunostaining of GM1 ganglioside and Galgt1 protein in 6-week-old *mdx* and *Galgt1* ^*−/−*^ *mdx* skeletal muscle. **(B)** qRT-PCR comparison of *Galgt1* gene expression in WT and *mdx* skeletal muscle at 6 weeks and 3 months of age. **(C)** GM1 and secondary antibody (control) staining in muscle sections obtained from patients with Duchenne muscular dystrophy (DMD) or from otherwise normal human muscle. Errors in **(B)** are SEM averaged from six muscles per condition with two to three measures per muscle. ***P < 0.001, Bar is 50 μm.

We next assessed Galgt1 and GM1 expression in dystrophin-deficient mouse (*mdx*) and human (DMD) skeletal muscles, where chronic cycles of muscle regeneration occur (Figure 2). In *mdx* skeletal muscle, expression of Galgt1 protein and GM1 ganglioside were increased in mononuclear cells (Figure 2A), much as we had previously observed in CTX-induced wild type muscle (Figure 1C). Staining for Galgt1 and GM1 was absent in *Galgt1*^*−/−*^*mdx* muscles, which were used to demonstrate antibody specificity. *Galgt1* gene expression was upregulated 16-fold in 6-week-old mdx muscle and fivefold in 3-month-old mdx muscle relative to age-matched WT muscles (Figure 2B). The increased expression of GM1 seen in mdx mice also occurred in muscles of DMD patients when compared to otherwise normal human controls (Figure 2C). In all cases, background staining with secondary antibody alone (control) showed no significant staining (Figure 2C). Unfortunately, mRNA extracted from these human muscle samples was of insufficient quality to allow qRT-PCR measures of *Galgt1* gene expression.

### *Galgt1*-deficient muscles show altered regeneration in acute and chronic muscle injury models

**Figure 3:**
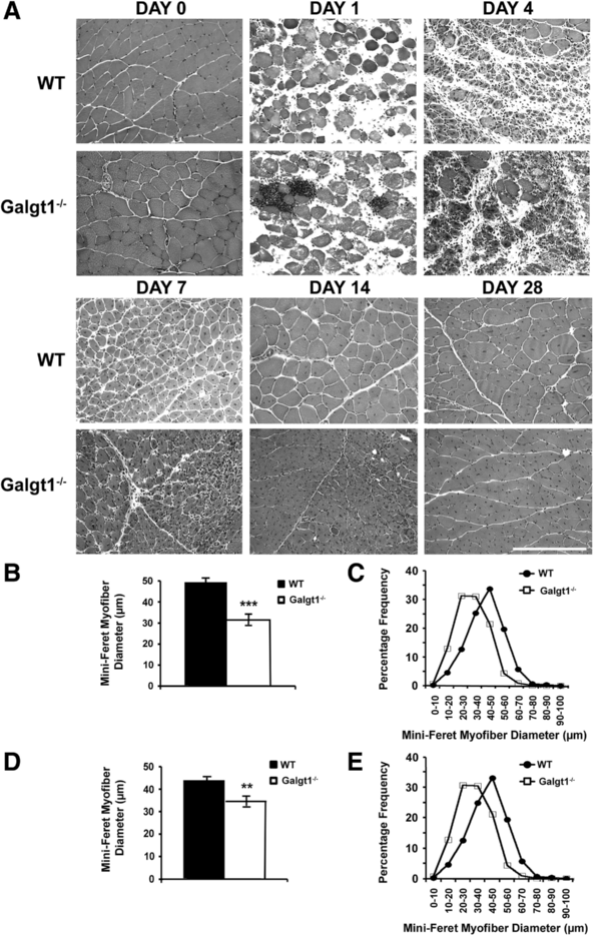
**Muscle regeneration after cardiotoxin-induced injury in normal and** ***Galgt1*** **-deficient mouse muscle. (A)** Hematoxylin and Eosin staining of wild type (WT) and *Galgt1*-deficient (*Galgt1* ^*−/−*^) mouse muscle at various days after cardiotoxin-induced injury. **(B)** Average myofiber diameter of WT and *Galgt1* ^*−/−*^ gastrocnemius muscle and **(C)** distribution of myofiber diameters at Day 14 after cardiotoxin-induced injury. **(D)** Average myofiber diameter of WT and *Galgt1* ^*−/−*^ gastrocnemius muscle and **(E)** distribution of myofiber diameters at Day 28 after cardiotoxin-induced injury. Errors in **(B)** and **(D)** are SEM from six muscles per condition with 1,600–3,200 total myofibers counted per condition. **P < 0.01, ***P < 0.001, Bar is 200 μm.

To test whether increased *Galgt1* expression was important for muscle regeneration, we compared regeneration in WT and *Galgt1*-deficient (*Galgt1*^*−/−*^) mice in response to acute muscle injury induced by CTX (Figure 3). In response to CTX, muscles of *Galgt1*^*−/−*^ mice were still able to regenerate, showing that *Galgt1* is not essential for skeletal muscle regeneration. There was, however, a significant difference in the extent of regeneration between time-matched CTX-treated *Galgt1*^*−/−*^ and WT muscles (Figure 3A). The average myofiber diameter of *Galgt1*^*−/−*^ muscles was significantly smaller than WT on Day 14 (Figure 3B) and at Day 28 (Figure 3D) after CTX. A distribution curve of muscle diameters was plotted to see the range and frequency of muscle sizes in WT and *Galgt1*^*−/−*^ mice. At Day 14 (Figure 3C) and at Day 28 (Figure 3E) post-CTX, the Mini-Feret diameters of Galgt1^−/−^ myofibers were smaller when compared to WT. There was no any evidence of muscle hypertrophy in *Galgt1*^*−/−*^ muscles at any time after CTX. Thus, although the absence of *Galgt1* did not abolish muscle regeneration, it did alter the rate and/or extent to which muscle regeneration occurred.

**Figure 4:**
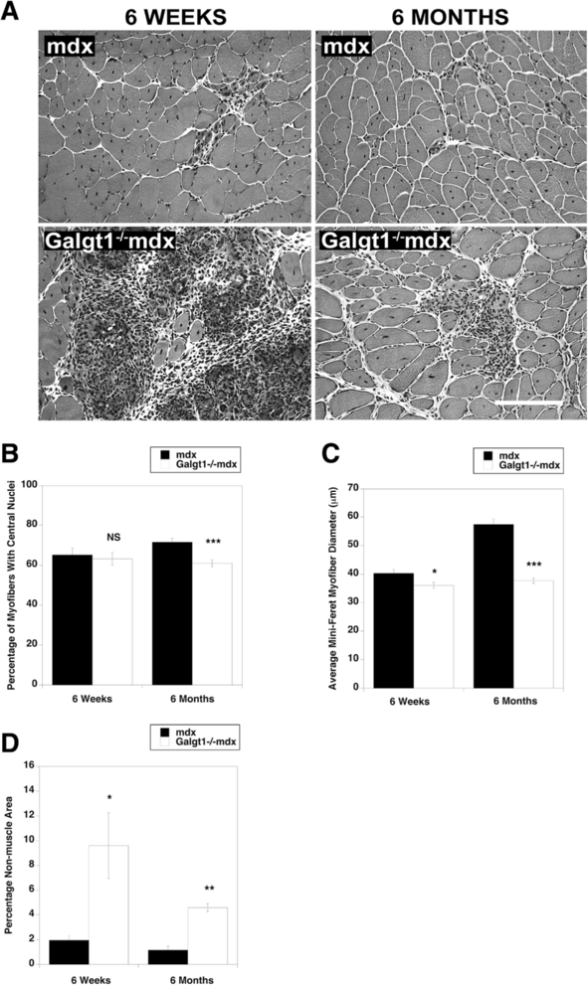
**Muscle phenotypes in young and old** ***mdx*** **and** ***Galgt1*** **-deficient** ***mdx*** **muscles. (A)** Hematoxylin and eosin staining of cross sections of gastrocnemius muscle in young (6 weeks) and old (6 months) *mdx* and *Galgt1* ^*−/−*^ *mdx* animals. **(B)** Average percentage central nuclei in *mdx* and *Galgt1* ^*−/−*^ *mdx* gastrocnemius at 6 weeks and 6 months of age. **(C)** Average Mini-Feret myofiber diameters in *mdx* and *Galgt1* ^*−/−*^ *mdx* gastrocnemius muscle at 6 weeks and 6 months of age. **(D)** Average percentage of non-muscle area present in muscle sections in *mdx* and *Galgt1* ^*−/−*^mdx muscles at 6 weeks and 6 months of age. Errors in **(B)**–**(D)** are SEM from six muscles per condition with 1,600–3,200 total myofibers counted per condition. *P < 0.05, **P < 0.01, ***P < 0.001, *NS* not significant, Bar is 50 μm.

To assess whether regeneration was altered by the loss of *Galgt1* in a chronic injury model, we interbred *mdx* and *Galgt1*^*−/−*^ mice to obtain double knockout (*Galgt1*^*−/−*^*mdx*) mice (Figure 4). In young *Galgt1*^*−/−*^*mdx* mice (6 weeks), a time at which muscle damage in *mdx* mice is more severe [21], there was a marked increase in necrotic areas containing very high-density mononuclear cell infiltrates when compared to *mdx* littermates (Figure 4A). This increase was still present, though less pronounced, in *Galgt1*^*−/−*^*mdx* animals at 6 months of age (Figure 4A). Interestingly, the percentage of myofibers with centrally located nuclei, a measure of the extent of muscle regeneration, was not significantly changed at 6 weeks of age but was changed at 6 months of age in *Galgt1*^*−/−*^*mdx* mice compared to age-matched *mdx* controls (Figure 4B). The average myofiber diameter, by contrast, was significantly changed in *Galgt1*^*−/−*^*mdx* muscles at both ages (Figure 4C). Similarly, at both 6 weeks and 6 months, the percentage area within the muscle not containing skeletal myofibers, a measure of muscle damage and wasting, was increased in *Galgt1*^*−/−*^*mdx* mice compared to *mdx* (Figure 4D). The fact that *mdx* mice show more profound muscle damage in the early interval at 4–6 weeks of age may explain some of the age-related differences in these measures, but the results with *Galgt1*^*−/−*^*mdx* mice reiterate the finding of reduced muscle growth found with CTX injury in *Galgt1*^*−/−*^ mice and have additionally the finding of increased muscle tissue loss, a finding not found in CTX-treated *Galgt1*^*−/−*^ muscles. Cumulatively, these data support the notion that loss of *Galgt1* alters the kinetics and/or the extent of muscle regeneration both in acute and chronic models of muscle damage.

### *Galgt1* is overexpressed in muscle cells following muscle injury

**Figure 5:**
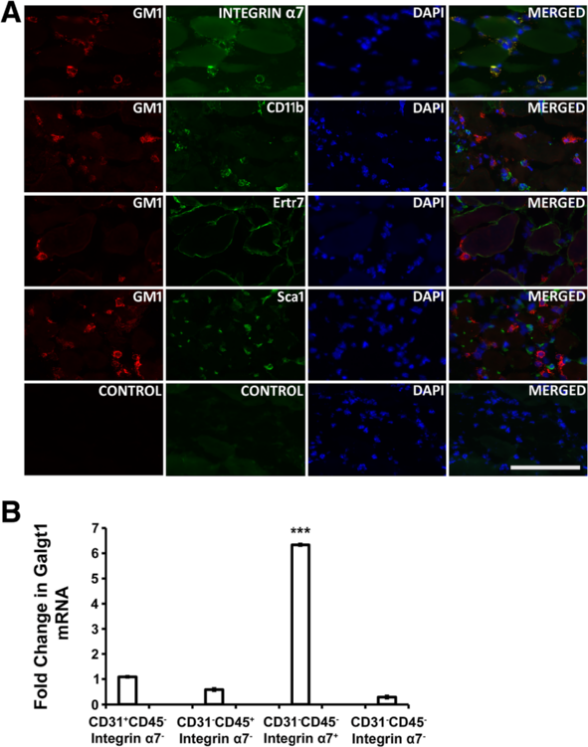
***Galgt1*** **gene expression is elevated in muscle cells during muscle regeneration. (A)** Immunostaining with antibodies to GM1 (red) and antibodies to cell-specific markers (green) for muscle cells (integrin α7), immune cells (CD11b), fibroblasts (Ertr7), or mesenchymal stem cells (Sca1), with DAPI (blue) nuclear stain, 1 day after cardiotoxin injection into the gastrocnemius muscle. Three-color merged images are shown on the right. **(B)** qRT-PCR comparison of *Galgt1* gene expression in FACS-sorted cell populations after 1 day of cardiotoxin-induced injury compared to the same populations sorted from untreated controls. ***P < 0.001 (comparing CD31^−^CD45^−^integrin α7^+^ to any of the other shown conditions). Errors in **(B)** are SEM averaged from four experiments per condition with two to three measures per muscle. Bar is 50 μm.

We next used FACS to determine which intramuscular mononuclear cell types overexpress *Galgt1* during muscle regeneration in response to CTX (Figure 5). To do this, WT skeletal muscles were injected with CTX and mononuclear cells were sorted by FACS 1 day later and compared to cells isolated and sorted from non-CTX-injected controls (vehicle only). In each instance, cells were first gated for Sca1 expression (Sca1^+^ and Sca1^−^). Sca1 is a marker for mesenchymal stem cells. Sca1^+^ and Sca1^−^ cell populations were then sorted and collected for Sca1^+^CD31^+^, Sca1^+^CD45^+^, Sca1^+^CD31^−^CD45^−^, Sca1^−^CD31^+^, Sca1^−^CD45^+^, and Sca1^−^CD31^−^CD45^−^ (data not shown). CD31 is a marker for endothelial cells and CD45 is a marker for immune cells. The mRNA from various CTX-treated fractions was analyzed for *Galgt1* expression using qRT-PCR and compared to the same fractions isolated from non-CTX-treated controls. Of these, *Galgt1* expression was only significantly increased in the Sca1^−^CD31^−^CD45^−^ CTX-treated fraction (data not shown). Since the major cell population in this fraction would be muscle cells (satellite cells and myoblasts) and intramuscular fibroblasts, cells were next sorted for CD31^+^, CD45^+^, CD31^−^CD45^−^integrin α7^+^, and CD31^−^CD45^−^integrin α7^−^ cells to separate the muscle cell population (CD31^−^CD45^−^integrin α7^+^) from the endothelial (CD31^+^), immune cell (CD45^+^), and fibroblast-containing cell (CD31^−^CD45^−^integrin α7^−^) populations. The CD31^−^CD45^−^integrin α7^+^ population was the only cell type that showed an increase in *Galgt1* expression relative to non-CTX-treated muscle, with over a sixfold average increase (Figure 5B). None of the other three cell types showed any increase in *Galgt1* expression in cells taken from CTX muscles when compared to non-CTX-treated controls. To verify this result, we double immunostained for GM1 with integrin α7 (muscle cell marker), CD11b (immune cell marker, including macrophages), Ertr7 (fibroblast marker), or Sca1 (mesenchymal cell marker) (Figure 5A). GM1 staining was localized in cells also expressing integrin α7, but did not co-localize with cells expressing Sca1, Ertr7, or CD11b. Similar results were obtained for Galgt1 protein-double immunostaining (data not shown). GM1 staining sometimes appeared in a punctate pattern, which may be consistent with non-uniform membrane expression, such as one might observe in lipid rafts. These data demonstrate that muscle cells have increased levels of Galgt1 and GM1 and largely contribute to the marked elevation of *Galgt1* expression in regenerating muscle.

### Regenerating *Galgt1*^*−/−*^ muscle has altered expression of satellite and myoblast cell markers

**Figure 6:**
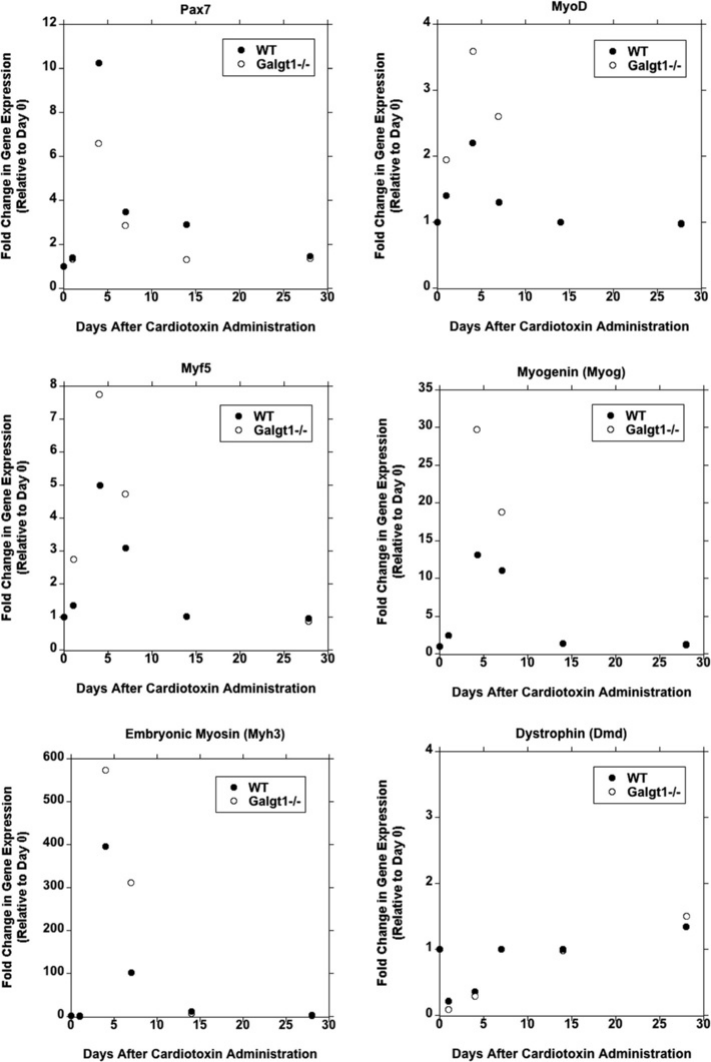
**Altered gene expression of satellite cell, myoblast, and early myofiber markers in regenerating** ***Galgt1*** ^***−/−***^ **muscle.** Wild type (WT) and *Galgt1*-deficient (*Galgt1* ^*−/−*^) muscles were injected with cardiotoxin and gene expression measured at Day 1, 4, 7, 14, and 28, relative to control untreated muscle (Day 0). Gene expression was compared for Pax7, a satellite cell marker; MyoD, Myf5, and Myogenin (*Myog*), myoblast cell markers; Myh3 (embryonic myosin), a marker for early differentiating myofibers; and Dystrophin (*Dmd*), a marker for mature myofibers. Data represent averages of three to four experiments per condition, with two to three measures per point.

As we had identified muscle cells as the major cell type showing increased *Galgt1* expression during CTX-induced regeneration (Figure 5), we next compared gene expression for a marker of satellite cells (Pax7), several markers of myoblasts (MyoD, Myf5, and Myogenin), a marker of early differentiating myofibers (embryonic myosin, Myh3), and a marker of mature myofibers (dystrophin) at various times after CTX-induced injury (Figure 6). Pax7 expression was reduced in *Galgt1*-deficient muscles at 4, 7, and 14 days post-CTX injury relative to WT (*P* < 0.001 at Day 4 and 14). By contrast, expression of MyoD and Myf5 was elevated at Days 1, 4, and 7 after CTX, as was Myogenin at Days 4 and 7 (*P* < 0.001 for all). Expression of embryonic myosin (Myh3) was also elevated in *Galgt1*^*−/−*^ muscles at Days 4 and 7 (*P* < 0.001 for both), while expression of dystrophin was not changed at these times. These data suggest that while *Galgt1* expression is elevated in mononuclear muscle cells after CTX-induced injury, this differentially affects the expression of markers for satellite cells and myoblasts. Such data would be consistent with an over-induction of myoblast differentiation at the expense of self-renewing satellite cells in regenerating *Galgt1*^*−/−*^ muscle. Several additional experiments also pointed to this conclusion.

### Myoblast cell fusion is accelerated in time but reduced in extent in the absence of *Galgt1*

**Figure 7:**
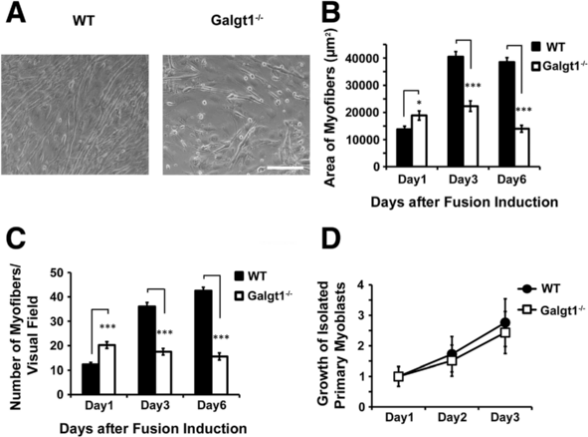
**Altered differentiation of cultured** ***Galgt1*** ^***−/−***^ **muscle cells. (A)** Phase-contrast images of cultured confluent wild type (WT) and *Galgt1*-deficient (*Galgt1* ^*−/−*^) primary muscle cells 6 days after placement in myofiber differentiation media. **(B)** Area of myofibers per unit area of the visual field at 1, 3, or 6 days after addition of differentiation media. **(C)** Number of myofibers present per unit area of the field of view at 1, 3, or 6 days after addition of differentiation media. **(D)** Growth rate of isolated primary muscle cells cultured in growth media. Errors are SEM for *n* = 6 experiments in **(B)**–**(D)**, with three to six replicates per data point. *P < 0.01, ***P < 0.001, Bar is 50 μm.

We next performed studies on isolated muscle cells purified from WT and *Galgt1*-deficient (*Galgt1*^*−/−*^) muscles (Figure 7). Isolated cells were pre-plated to remove intramuscular fibroblasts and to enrich for muscle cells. Cells were grown to confluency and then switched to low-serum-containing media to induce myoblast fusion into myofibers, as previously described [61]. WT muscle cells formed long, thick, and abundant myofibers after 6 days in fusion media, whereas *Galgt1*^*−/−*^ muscle cells formed less abundant and often shorter and smaller myofibers (Figure 7A–C). Interestingly, on Day 1, after placement of confluent cultures in fusion media, the number of myofibers (Figure 7C) and the area of fused myofibers per visual field (Figure 7B) were significantly higher in cells isolated from *Galgt1*^*−/−*^ muscle when compared to WT. On Day 3 and Day 6, however, *Galgt1*^*−/−*^ cultures showed significantly reduced numbers of myofibers with smaller overall area (Figure 7B, C). To ensure that this difference in fusion between WT and *Galgt1*^*−/−*^ myoblasts was not due to difference in myoblast growth, we measured the growth rate of WT and *Galgt1*^*−/−*^ cells cultured in growth media and found no significant difference (Figure 7D). Assessment of myoblast migration in a classical scratch assay also showed no significant change (data not shown). These data are consistent with the premature differentiation of cultured muscle cells into myofibers in *Galgt1*^*−/−*^ muscles.

### Increased apoptosis in satellite cells in *Galgt1*^*−/−*^ muscle *in vitro* and *in vivo*

**Figure 8:**
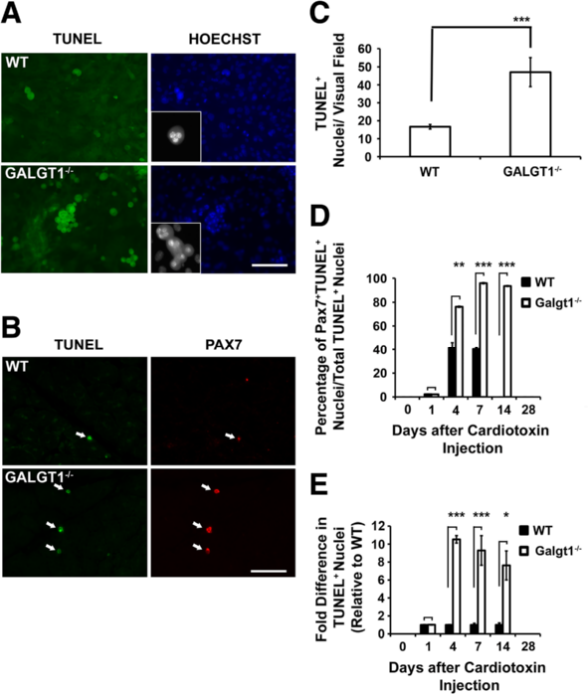
**Increased apoptosis of** ***Galgt1*** **-deficient muscle cells** ***in vitro*** **and** ***in vivo*** **. (A)** TUNEL and Hoechst co-staining on primary muscle cell cultures isolated from wild type (WT) and *Galgt1*-deficient (*Galgt1* ^*−/−*^ *)* skeletal muscle 1 day after placement in differentiation media. **(B)** Double immunostaining for Pax7 and TUNEL of *Galgt1* ^*−/−*^ skeletal muscle after CTX injection. Apoptotic nuclei are visualized with green fluorescent TUNEL staining, with Pax7^+^ staining in red. Arrows indicate regions with co-staining. **(C)** Quantification of TUNEL^+^ nuclei per visual field in WT and *Galgt1* ^*−/−*^ myoblast cultures 1 day after addition of differentiation media. **(D)** Percentage of cells positive for both Pax7 and TUNEL, relative to all TUNEL^+^ cells, at various days after cardiotoxin injection of wild type (WT) and *Galgt1*-deficient (*Galgt1* ^*−/−*^) muscle. **(E)** Fold difference in TUNEL^+^ nuclei in the *Galgt1* ^*−/−*^ skeletal muscle, relative to WT, at various days after CTX injection. Errors in **(C)** are SEM for *n* = 6 experiments with three to four replicates per condition and SEM for *n* = 6 experiments in **(D)** and **(E)** with three to four sections analyzed per muscle. *P < 0.05, **P < 0.01, ***P < 0.001. Bar is 50 or 12.5 μm (insert).

One possible cause of reduced myofiber formation over time would be an increase in satellite cell or myoblast cell death during the induction of differentiation. To investigate whether *Galgt1*-deficient muscle cells were more susceptible to apoptosis during the induction of differentiation, we performed TUNEL staining on cultured muscle cells *in vitro* as well as in CTX-treated skeletal muscle *in vivo* (Figure 8). Isolated muscle cells from *Galgt1*^*−/−*^ muscles showed a threefold increase in TUNEL staining relative to cells isolated from WT muscles after addition of differentiation media (Figure 8A, C). Such TUNEL-positive *Galgt1*^*−/−*^ cells showed classical signs of apoptosis, including increased chromatin condensation and nuclear fragmentation (Figure 8A). The permeabilization conditions used here led to significant cytoplasmic TUNEL staining in some cells. When tissue sections from CTX-injected muscles were stained for TUNEL, there was a 7.6–10.5-fold increase in the number of stained cells in *Galgt1*^*−/−*^ muscles compared to WT at Days 4, 7, and 14 post-injury (Figure 8B, E). To assess if Pax7^+^ satellite cells had increased apoptosis, we co-stained cells in WT and *Galgt1*^*−/−*^ muscles for TUNEL and Pax7 (Figure 8D). At Days 4 and 7 in WT muscle, a significant percentage of TUNEL-positive apoptotic cells, about 40%, were Pax7-positive. The overall number of such TUNEL-positive cells, however, was quite small (Figure 8E). By contrast, in *Galgt1*^*−/−*^ muscle, the vast majority (93%–96% at Days 7 and 14) of TUNEL-positive cells were also Pax7-positive, and the number of TUNEL-positive cells was significantly increased relative to WT at Days 4, 7, and 14. It is important to point out that there were still many Pax7^+^ cells that were not stained for TUNEL in *Galgt1*^*−/−*^ CTX-treated muscles and that regeneration likely progressed due to the presence of these remaining, non-dying, satellite cells. These data demonstrate that the majority of muscle cells undergoing apoptosis during regeneration in *Galgt1*^*−/−*^ muscles are Pax7-positive satellite cells and suggest that *Galgt1* modulates satellite cell survival during muscle regeneration.

## Discussion

Here we have described a new role for complex gangliosides made by *Galgt1*, a gene encoding a β1,4GalNAc glycolipid glycosyltransferase, in muscle regeneration. *Galgt1* expression was dramatically elevated in the first few days after induction of acute muscle regeneration by cardiotoxin and was elevated in *mdx* muscles, which undergo chronic regeneration as well. During regeneration, elevated *Galgt1* expression was identified in muscle cells, but not in intramuscular fibroblasts, immune cells, or mesenchymal stem cells. Moreover, studies of muscle cells isolated from normal and *Galgt1*-deficient muscles demonstrated that *Galgt1* is required for proper muscle cell differentiation and survival; regenerating *Galgt1*^*−/−*^ muscles showed a deficit in gene expression for the satellite cell marker Pax7 and increased gene expression for myoblast markers MyoD, Myf5, and Myogenin, cultured muscle cells from *Galgt1*^*−/−*^ mice showed premature fusion into myofibers which resulted in less robust myofiber formation over time, and regenerating muscles from *Galgt1*^*−/−*^ mice showed an order of magnitude increase in the apoptosis of Pax7-positive cells during the regeneration process. The end result, both for acute and chronic muscle regeneration models, was a deficit in regeneration with increased populations of smaller caliber myofibers. In *Galgt1*^*−/−*^dystrophin-deficient (*mdx*) muscles, this was also coupled with increased loss of muscle tissue. Further work will be needed to more carefully analyze the possible mechanisms of action, but this study clearly shows that *Galgt1* plays a role in the regenerative process in skeletal muscle and identifies complex gangliosides made by *Galgt1* as potential targets for therapeutic interventions to stimulate aspects of muscle regeneration.

Our findings are consistent with previous studies implicating other types of glycans, including glycosaminoglycans and N-linked glycans, in satellite cell function during muscle regeneration. The results reported here showing reduced Pax7 expression and increased MyoD expression in *Galgt1*^*−/−*^ muscle, for example, are similar to the studies of Denis and colleagues on *Mgat5*-deficient mice, which had reduced satellite cell number with a bias towards increased MyoD expression at the expense of Pax7 [70]. *Mgat5* affects the formation of β1,6-GlcNAc-branched N-linked glycans and would not directly affect the biosynthesis of gangliosides made by *Galgt1*; nevertheless, it is possible that these pathways might intersect through a less-direct mechanism. Emerson and colleagues have shown that inhibition of heparin sulfate 6-O-endosulfatases (Sulfs) in *Sulf1*/*Sulf2* double knockout mice increases the number of Pax7-positive satellite cells as well as increasing fibroblast growth factor (FGF) signaling [43]. Ai and colleagues have further shown a complex differential regulation of canonical and non-canonical Wnt signaling pathways in *Sulf1/2* double mutant mice, with a bias towards premature differentiation of satellite cells and formation of myofibers with smaller overall area after cardiotoxin-induced injury [44], akin to what we observed in these studies in *Galgt1*-deficient mice. Work by Olwin, Brandan, Velleman, and others suggests that mice or cells lacking the proteoglycans Syndecan-3 or Syndecan-4 show impairment of satellite cell division and/or differentiation, including altered induction of MyoD expression and localization as well as impaired myofiber formation [38,39,42,71,72]. These syndecans could impact signaling of multiple regeneration signaling pathways, including NotchR, FGF, and Wnt [42]. Gangliosides, through their ability to orient proteins in lipid rafts on the cell membrane, might interact with any or all of these other glycan-dependent pathways to regulate their ability to govern various signaling functions in muscle cells. Future work will be required to delineate the possible pathways by which complex gangliosides, N-linked glycans, and glycosaminoglycans might intersect to modulate these vital regenerative signaling pathways.

A number of studies support such roles for complex gangliosides in muscle cell function. Gangliosides are regulated differentially through the various stages of muscle cell differentiation [73-75]. Since GD1a and GM1 expression are both increased in cultured myoblasts just before fusion [75], and as they both require *Galgt1* for their biosynthesis [56], their elevated expression likely arises from increased *Galgt1* gene expression. GM1 has also been shown to be present on the lamellipodia of myoblasts, and affecting the clustering and dispersal of lipid rafts has been shown to be important for myoblast fusion [76-78]. Studies on the fusion-resistant fu-1 rat myoblast cell line have demonstrated that there is a consistent decrease in the levels of glycosphingolipids in these cells and that they have lost the ability to differentiate and express the markers of differentiation [75,79,80]. Glycosylation inhibitors like tunicamycin also impair myoblast cell fusion [81], and no myoblast fusion is observed in certain lectin-resistant muscle cell mutants [79,80,82]. Many other studies suggest that gangliosides can alter intracellular signaling by kinases [49,50,52,53,83,84], including kinases known to affect muscle regeneration such as Akt [85,86] and mTOR [87]. Modulation of these and other signaling pathways would also impact muscle cell growth or differentiation. One current obstacle to muscle cell therapies is the inefficient muscle engraftment of transplanted stem or induced pluripotent stem cells (iPS) [88]. This too would be an avenue worth exploring as regards *Galgt1* function as a potential stimulator of muscle regeneration, as it appears unlikely that the logarithmic increase in *Galgt1* gene expression seen in muscle cells a day after injury *in vivo* would be maintained once these cells were isolated and amplified for prolonged periods in culture.

## Conclusions

This work demonstrates a role for *Galgt1* during muscle regeneration. *Galgt1* gene expression is dramatically elevated in both acute and chronic mouse models of muscle injury, as is GM1, a ganglioside that requires *Galgt1* activity for its biosynthesis. Loss of *Galgt1* in mice leads to impaired muscle regeneration in both acute and chronic muscle injury models, and loss of *Galgt1* is coupled to premature differentiation and increased apoptosis of satellite cells *in vitro* and *in vivo*. These data support the notion that complex gangliosides generated by *Galgt1* play modulatory roles during muscle regeneration and might be therapeutic targets in myopathies where muscle regeneration is impaired or insufficient.

## Abbreviations

CTX: Cardiotoxin
DMD: Duchenne muscular dystrophy
WT: Wild type
TUNEL: Terminal deoxynucleotidyl transferase
TA: Tibialis anterior
NMJ: Neuromuscular junction
FACS: Fluorescence-activated cell sorting
iPS: Induced pluripotent stem cells
BSA: Bovine serum albumin
PBS: Phosphate-buffered saline
H&E: Hematoxylin and eosin
qRT-PCR: quantitative Real-time Polymerase Chain Reaction
